# Alterations of Brain Functional Architecture Associated with Psychopathic Traits in Male Adolescents with Conduct Disorder

**DOI:** 10.1038/s41598-017-11775-z

**Published:** 2017-09-12

**Authors:** Weidan Pu, Qiang Luo, Yali Jiang, Yidian Gao, Qingsen Ming, Shuqiao Yao

**Affiliations:** 1 Medical Psychological Center, the Second Xiangya Hospital, Central South University, Changsha, P.R. China; 2 Medical Psychological Institute of Central South University, Changsha, P.R. China; 3 School of Life Sciences, Fudan University, Shanghai, P.R. China; 4 Institute of Science and Technology for Brain-Inspired Intelligence, Fudan University, Shanghai, P.R. China

## Abstract

Psychopathic traits of conduct disorder (CD) have a core callous-unemotional (CU) component and an impulsive-antisocial component. Previous task-driven fMRI studies have suggested that psychopathic traits are associated with dysfunction of several brain areas involved in different cognitive functions (e.g., empathy, reward, and response inhibition etc.), but the relationship between psychopathic traits and intrinsic brain functional architecture has not yet been explored in CD. Using a holistic brain-wide functional connectivity analysis, this study delineated the alterations in brain functional networks in patients with conduct disorder. Compared with matched healthy controls, we found decreased anti-synchronization between the fronto-parietal network (FPN) and default mode network (DMN), and increased intra-network synchronization within the frontothalamic–basal ganglia, right frontoparietal, and temporal/limbic/visual networks in CD patients. Correlation analysis showed that the weakened FPN-DMN interaction was associated with CU traits, while the heightened intra-network functional connectivity was related to impulsivity traits in CD patients. Our findings suggest that decoupling of cognitive control (FPN) with social understanding of others (DMN) is associated with the CU traits, and hyper-functions of the reward and motor inhibition systems elevate impulsiveness in CD.

## Introduction

Conduct disorder (CD) is a serious mental disorder of childhood and adolescence that is characterized by persistent behavioral pattern in which the basic rights of others, or rules or laws are violated^1^. CD patients with elevated psychopathic traits have more severe and stable antisocial behavior, and poorer response to treatment^2^. Such psychopathic traits have a core callous-unemotional (CU) component (lack of guilty and empathy) and an impulsive-antisocial component^3^. However, the neural underpinnings of these components in CD patients remain elusive.

Previous studies have revealed that conduct problems are associated with abnormal activities in brain regions implicated in a wide range of socio-cognitive functions ranging from motivation^4^, inhibition^5^, reinforcement learning^3, 6–9^, to empathy^10^. Most prominently, those focusing on empathy and emotional face recognition have consistently demonstrated a close relationship between CU traits and reduced response of amygdale, ventral medial prefrontal cortex (VmPFC) and insula to distress cues^6, 11–13^, particularly for fear and pain of others. The reduced response of those brain regions to other’s distress cues is considered to be critical for the lack of empathy in the CD patients, which may lead to reduced aversion for actions that harm others. Interestingly, a recent fMRI study found that the anti-correlation between a fronto-parietal control network (FPN) and the default mode network (DMN) during a social working memory task was negatively correlated with empathy in healthy subjects^14^, and another study using resting-state fMRI approach showed a significant association between the FPN-DMN anti-correlation and trait emotional intelligence^15^. These observations raise a possibility that the psychopathic traits in psychiatric disorders (e.g., CD) may be not only related to the dysfunction in specific brain regions, but also affected by the dynamics between core brain networks.

Unlike the CU traits, very few studies have been performed to investigate the neural correlates of impulsivity in CD patients. However, valuable insights into the neurophysiology of impulsivity have been provided by studies in healthy controls and other mental disorders involved in psychopathic impulsivity such as ADHD and substance abuse^16–19^, which consistently document a close relationship between impulsivity and a fronto-striatal circuit (mainly composed of VmPFC, insula and striatum) subserving reward. Dysfunction of this fronto-striatal circuit has also been observed in CD patients while attending to reinforcement learning and reversal learning tasks^6^. Another brain network involved in impulsivity is composed of inferior frontal gyrus, supplementary motor area (SMA), pre-motor, and parietal cortex, which is consistently observed critical for response inhibition^20^. Abnormal activations in the SMA, prefrontal and parietal cortices during response inhibition task have been identified in CD patients^5, 21^, suggesting the involvement of inhibition-related network in the neuropathology of CD impulsivity traits.

As mentioned above, task-driven fMRI researches have helped to elucidate several potential neural substrates of psychopathic traits in CD. However, constrained by limited cognitive process that one task can probe, our understanding of the neural correlates of psychopathic traits with the alteration of brain functional architecture in CD is still fragmented. Moreover, psychopathic traits, like the normal personality traits, have a complex construct which is influenced by multiple systems^3, 17^. It is possible that individual differences in psychopathic traits are associated with broader patterns of global information processing that may extend beyond the previously identified circumscribed brain regions by specific task-driven fMRI data. Instead, the resting-state fMRI (rsfMRI) offers us a unique opportunity to appreciate the brain-wide functional architecture. Of note, such an approach has exerted valuable insights into the neural underpinnings of several normal and abnormal personality traits such as extraversion, neuroticism and borderline personality disorder^22–26^. In this study, applying a holistic brain-wide functional connectivity analysis on resting-state fMRI data, we aimed at associating abnormalities in intrinsic brain networks of adolescents with CD with their core psychopathic traits.

We firstly hypothesized that neural modules relevant to empathy (e.g., amygdala)^27^ would be impaired and associated with CU traits, while the disrupted fronto-striatal circuit responsible for reward^3^ and the inhibition-relevant circuit would be related to impulsivity in CD patients. Secondly, given the evidence of the association between the FPN-DMN anti-correlation and empathy in healthy subjects^15^, we anticipated that FPN-DMN dynamic interaction might be impaired and associated with the CU traits (lack of empathy) in CD patients.

## Methods

### Ethical Statement

All participants and their parents gave their written informed assent and consent to participate in our study after detailed description of the risks and benefits. The study was approved by the ethics committee of the Second Xiangya Hospital, Central South University. All the subsequent research analyses were carried out in accordance with the approved guidelines.

### Participants

A total of forty-two male adolescents with CD were recruited from the Second Xiangya Hospital of Central South University, Changsha, China. Forty-one matched healthy volunteers were recruited from a regular school in the same city. All subjects were right-handed according to the Edinburgh Handedness Inventory^28^.

Two experienced psychiatrists over 10 years diagnosed CD independently based on the Structured Clinical Interview of the Diagnostic and Statistical Manual of Mental Disorders IV, Text Revision, Axis I Disorders–Patient Edition^29^. Psychiatrists rated each symptom item as absent (0), subclinical (1), or clinically present (2). We did not diagnose conduct disorder (CD) based solely on information from adolescents; we also interviewed parents to obtain detailed information. Psychiatrists made final judgments on the consistency of information provided by patients and parents.

For recruitment of healthy control (HC), two investigators gave a detailed explanation of the study aim and procedure to the headmaster and teachers of the target school in person. Upon obtaining permission from the school’s administration, students who matched CD subjects’ ages, sex and years of education were selected randomly from class rosters. Volunteers who agreed to be interviewed by the psychiatrists were assessed using the SCID-I/P and Wechsler Intelligence Scale for Children–Chinese revision (C-WISC)^30^. Information provided by HCs was verified by their parents as needed. No HC met the criteria for CD.

Exclusion criteria for subjects in both groups were: history of attention deficit hyperactivity disorder, oppositional defiant disorder, or any other psychiatric or emotional disorder; diagnosis with any pervasive developmental or chronic neurological disorder, Tourette’s syndrome, post-traumatic stress disorder, or obsessive compulsive disorder; persistent headache or head trauma; alcohol or substance abuse in the past year; contraindication to magnetic resonance imaging; and IQ ≤ 80 according to the C-WISC. All participants with CD were treatment naïve and fulfilled the criteria for adolescent-onset CD, demonstrating at least one sign of CD after 10 years of age.

Depression and anxiety severity were rated using the Chinese version of the Center for Epidemiologic Studies Depression Scale^31^ and the Multidimensional Anxiety Scale for Children^32^, respectively. The Chinese versions of the Subjective Socioeconomic Status Scale^33^ and the Strength and Difficulties Questionnaire (SDQ)^34^ were used to assess socioeconomic status and detect internalizing and externalizing problems, respectively. The Antisocial Process Screening Device (APSD) was used to assess CU traits^35^, and the Barratt Impulsiveness Scale (BIS)^36^ was also used to assess impulsivity. These scales and questionnaires have shown enough reliability and validity in our previous studies^21, 37–39^.

### fMRI data acquisition and preprocessing

Resting-state fMRI data were acquired using a Philips Gyroscan Achieva 3.0 Tesla MRI scanner in the axial location, with a gradient-echo-planar imaging sequence. Details of image acquisition and fMRI data preprocessing can be found elsewhere^40–42^ and in the Supplemental Materials (Text. S1).

### Group Independent Component Analysis (ICA)

Spatial ICA was conducted with resting-state fMRI data from all 83 participants using the Informix algorithm with the Group ICA of fMRI Toolbox (GIFT) software (Medical Image Analysis Lab, University of New Mexico, Albuquerque, NM, USA; http://icatb.sourceforge.net/). The exact pipeline for ICA has been applied in our prior works^43, 44^ and other studies^45–49^. Based on the aggregate dataset from all participants, data dimensionality (number of components) was estimated using the minimum description length criteria tool in GIFT^45, 48, 49^, which suggested that 30 is the optimal number of independent components (ICs). The dimensions of the functional data were then reduced using principal component analysis^45, 46^, followed by an independent component estimation that produced spatial maps and time courses with the infomax algorithm^46^. Estimated ICs at the group level (both spatial maps and time courses) were then back-reconstructed for each participant based on principal components analysis compression and projection^45, 47, 48^, yielding subject-specific spatial maps and time courses for each estimated component. This specific back-reconstruction feature of the GIFT algorithm allows analysis of all participants simultaneously as part of a large ICA group matrix^47^. For each IC, the time courses of each component therefore represented a pattern of synchronized brain activity, whose coherency pattern across voxels was represented in the associated spatial map. To display voxels relevant to a particular IC, the intensity values in each map were converted to z values^50^.

### Identifying resting state networks

To identify valid resting state networks (RSNs), network components were examined visually to detect obvious artifacts and correlated spatially with *a priori* probabilistic gray-matter, white-matter, and cerebrospinal-fluid templates using multiple regression. As suggested by Meda *et al*.^51^, components associated weakly (│β│ < 0.05) with gray matter and strongly (│β│ > 2) with white matter and cerebrospinal fluid were identified as artifacts. Statistical maps for components were created using voxel-wise one-sample *t*-tests, with a threshold of *p* < 0.05 and family-wise error (FWE) correction. Fifteen ICA components were regarded as noise, leaving 15 ICs as RSNs of interest, which were considered for further analysis (Supplemental Figure S1, Supplemental Table S1). We then related each identified network to the brain functional maps produced by Laird *et al*.^52^ using voxel-wise spatial correlation analysis to determine their likely functions (Supplemental Table S1). Finally, spatial maps of each of the 15 networks from the two groups were compared using the two-sample *t*-test and SPM5 software (http://www.fil.ion.ucl.ac.uk/spm5). To ensure that only highly connected regions were analyzed, we used an explicit mask created with a voxel-wise one-sample *t*-test (*p* < 0.05, FWE correction). The significance level for each network in between-group comparison was adjusted for *q* < 0.05 with false discovery rate (FDR) correction.

### Functional Network Connectivity (FNC)

Before FNC analysis, fMRI data were band-pass filtered with a Butterworth filter with cutoff frequencies of 0.008–0.15 Hz^51^ (http://mialab.mrn.org/software). FNC analysis was used to examine specific temporal correlations in a nonparametric pair-wise manner, with a maximal lagged correlation approach. Time courses of all ICA networks were initially interpolated to detect finer and sub-repetition time hemodynamic differences. Then, all 15 RSNs were paired with one another to yield a total of 105 pair-wise combinations. Pair-wise correlation coefficients (representing the magnitude/extent of network connection) from all groups were extracted into SPSS software (version 19.0; SPSS Inc., Chicago, IL, USA). Resulting correlation data were transformed to Fisher’s *z* values and subjected to two-sample *t*-tests with a significance threshold at *q* < 0.05 with FDR correction (This FDR correction is based on all uncorrected p values from 105 two-sample t-tests).

Since recent studies^53, 54^ have suggested that subtle head movement is an important confounding factor for fMRI functional connectivity analysis, we also measured the head motion at each time-point which was calculated as the frame-wise displacement (FD). The mean absolute FD between HC and CD did not differ significantly (Mean ± SD__HC_: 0.21 ± 0.17, Mean ± SD__CD_: 0.19 ± 0.08, ns). After that, the mean absolute FD further entered into a contrast between HC and CD in a two-sample t-test to examine the group differences of intra-network or inter-network FC. The results were very similar to our prior findings without including FD as a nuisance covariate.

### Behavioral correlations of intra- and inter-network FC

For patients with CD, altered intra-network and inter-network FC within/between networks with mean loading coefficients showing a main effect of group difference were first extracted, and bivariate correlation with behavioral scale scores was then examined. For multiple testing, a powerful bootstrapping method^55^ was applied to reduce potential spurious findings; calculations were based on 5000 bootstrapped samples using biased corrected and accelerated 95% confidence intervals (CIs). Considering that the impulsivity measured by BIS screening is a continuous variable across normal adolescents and those with behavioral problems^36^, we also performed behavioral correlation analysis including all participants.

### Data Availability

The datasets generated during and/or analyzed during the current study are available from the corresponding author on reasonable request.

## Results

### Sample characteristics

Subjects’ demographic and clinical characteristics are summarized in Table 1. The two groups were well matched, with no significant difference in age, IQ, years of education, socioeconomic status, depression symptomology, or anxiety severity. SDQ total and conduct problems subscale scores, as well as APSD total and CU trait subscale scores, were significantly higher in the CD group than in the HC group. BIS total and motor and non-planning subscale scores were also significantly higher in the CD group than in the HC group, indicating that subjects with CD were more impulsive.

**Table 1:** Socio-demographical and Clinical Variables in Participants.

Variables	CD patients (Mean ± SD)	HC (Mean ± SD)	t value	p value
Age(year)	15.00 ± 0.99	15.16 ± 0.68	−0.75	0.45
IQ	103.79 ± 11.25	105.55 ± 5.70	−0.80	0.43
Education(year)	9.06 ± 0.78	9.13 ± 1.21	−0.76	0.39
SSS	6.39 ± 1.88	5.91 ± 1.35	1.06	0.30
CES-D	19.75 ± 8.10	17.34 ± 7.15	1.29	0.20
MASC	38.89 ± 18.13	36.34 ± 17.19	0.59	0.56
SDQ total score	14.22 ± 5.08	10.94 ± 3.59	3.04	0.003
SDQ conduct problem	3.97 ± 1.92	2.28 ± 1.20	4.41	<0.001
BIS impulsivity total score	75.39 ± 10.17	67.94 ± 7.15	3.42	0.001
BIS unplanned impulsivity	30.92 ± 4.34	27.68 ± 4.07	3.14	0.003
BIS motor impulsivity	26.28 ± 4.43	22.00 ± 3.44	4.37	<0.001
BIS attention impulsivity	18.19 ± 3.28	18.26 ± 2.87	0.08	0.93
APSD total score	16.03 ± 4.57	12.97 ± 3.36	3.14	0.003
APSD CU-traits	5.43 ± 1.74	4.28 ± 1.46	2.91	0.005

SSS: Subjective Socioeconomic Status Scale; CES-D: Center for Epidemiologic Studies Depression Scale; MASC: Multidimensional Anxiety Scale for Children; SDQ: the Strength and Difficulties Questionnaire; BIS: Barratt Impulsiveness Scale; APSD: Antisocial Process Screening Device; CU-traits: callous-unemotional traits.

### Altered intra-network and inter-network FC

**Figure 1:**
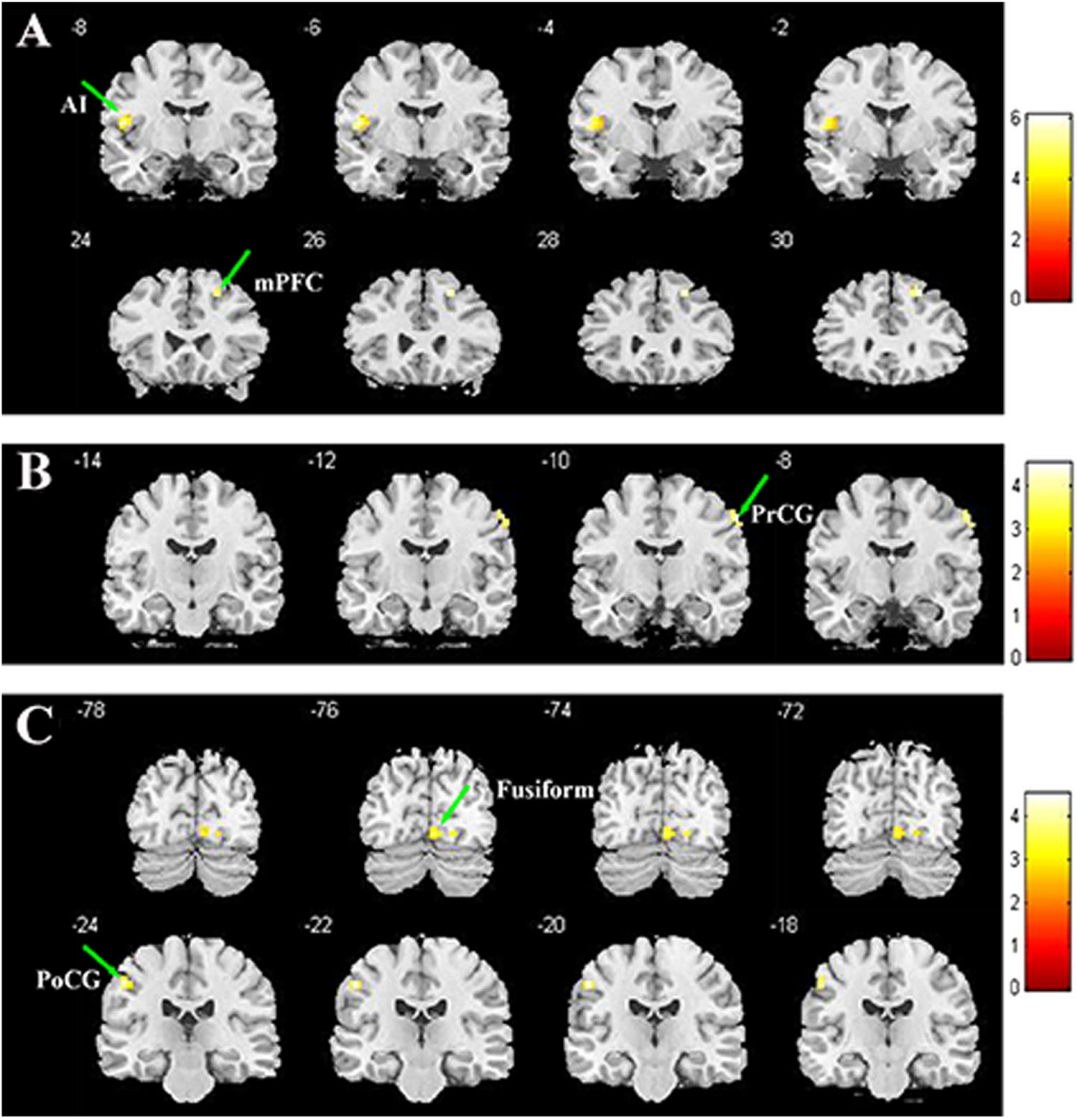
Altered Functional Connectivity within Brain Networks in Male Adolescents with Conduct Disorder. Compared with healthy controls (HC), patients with conduct disorder showed increased functional connectivity (FC) in the frontothalamic–basal ganglia (**A**), right frontoparietal (**B**), and temporal/limbic/visual (**C**) networks (q < 0.05, false discovery rate correction). All brain slices are in transverse view, with corresponding Montreal Neurological Institute slices (in millimeters).

Among the 15 ICs, three brain networks showed altered intra-network FC in patients with CD (*p* < 0.05, FDR correction; Fig. 1, Table 2). Relative to HCs, patients showed increased FC in the frontothalamic–basal ganglia (specifically located at the left anterior insula [AI, MNI = −42, −3, −9, cluster = 59, q < 0.001 with FDR correction] and right ventral medial prefrontal cortex [VmPFC, MNI = 21, 30, 48, cluster = 23, q = 0.042 with FDR correction]), right frontoparietal (right precentral gyrus [PrCG, MNI = 60, −9, 45, cluster = 15, q = 0.046 with FDR correction]), and temporal/limbic/visual (left fusiform, MNI = −33, −51, −21, cluster = 64, q = 0.002 with FDR correction, and postcentral gyrus [PoCG, MNI = −51, −21, 45, cluster = 27, q = 0.014 with FDR correction]) networks. Moreover, after integrating all 15 ICs into one component, a more stringent FDR correction (q < 0.05) was performed based on all of the voxels in this integrated component between two groups comparison. The results were similar to the prior findings showing that except for the VmPFC, other regions (insula, precental gyrus, fusiform and postcentral gyrus) were still survived.

**Table 2:** Altered Intra-network Functional Connectivity in Patients with Conduct Disorder as Compared to Healthy Controls.

IC Network	Regions(L/R)	Peak(x,y,z)	Cluster volume(mm^3^)^a^	t value^b^
Frontothalamic–basal ganglia (IC 11)	L.AI, extending to Rolandic operculum	−42, −3, 9	59	6.13**
	R.VmPFC	21, 30, 48	23	4.52*
right Fronto-parietal (IC17)	R.PrCG	60, −9, 45	15	4.41*
Temporal/limbic/visual (IC22)	L.fusiform	−33, −51, −21	64	5.41**
	L.PoCG	−51, −21, 45	27	5.00*

^a^Cluster volume column corresponds to regions within each network in Fig. 1. ^b^**q < 0.01, *q < 0.05, with FDR correction. AI: anterior insula; VmPFC: ventral medial prefrontal cortex; PrCG: precental gyrus; PoCG: postcentral gyrus; L: left, R: right.

**Figure 2:**
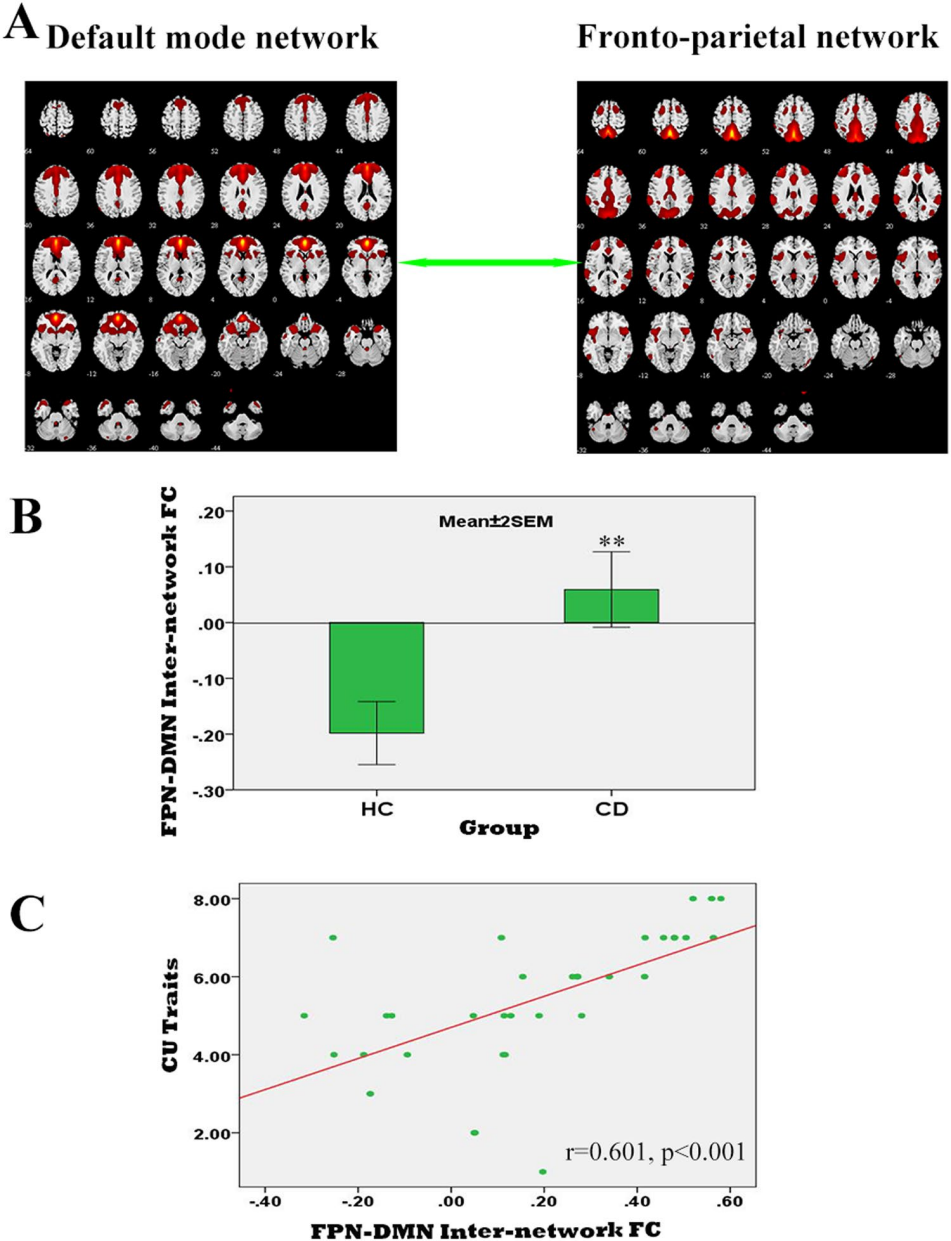
Altered Functional Connectivity between the Fronto-parietal and Default Mode Networks in Male Adolescents with Conduct Disorder. (**A**) Brain maps of the default mode network (DMN) and fronto-parietal network (FPN) derived from independent component analysis. (**B**) Anti-correlation between the DMN and FPN was reduced significantly in patients with conduct disorder (CD) compared with healthy controls (** q < 0.01, FDR correction). (**C**) Significant correlations between FPN-DMN inter-network FC and the callous-unemotional (CU) traits in patients with CD.

FNC analysis on the inter-FC between 15 brain networks revealed significantly increased FC only between the left fronto-parietal (FPN) and default mode network (DMN) in patients with CD compared with HCs (q < 0.05, FDR correction; Fig. 2A and B). Correlation coefficients between the FPN and DMN obtained by one-sample *t*-tests (*p* < 0.05, FWE correction) were significantly less than 0 in the HC group, suggesting a negative correlation, and greater than 0, but not significantly so, in patients with CD. We thus infer that the FPN–DMN anti-correlation was significantly reduced in patients with CD relative to HCs.

### Behavioral correlations of altered intra- and inter-network FC in patients with CD

**Figure 3:**
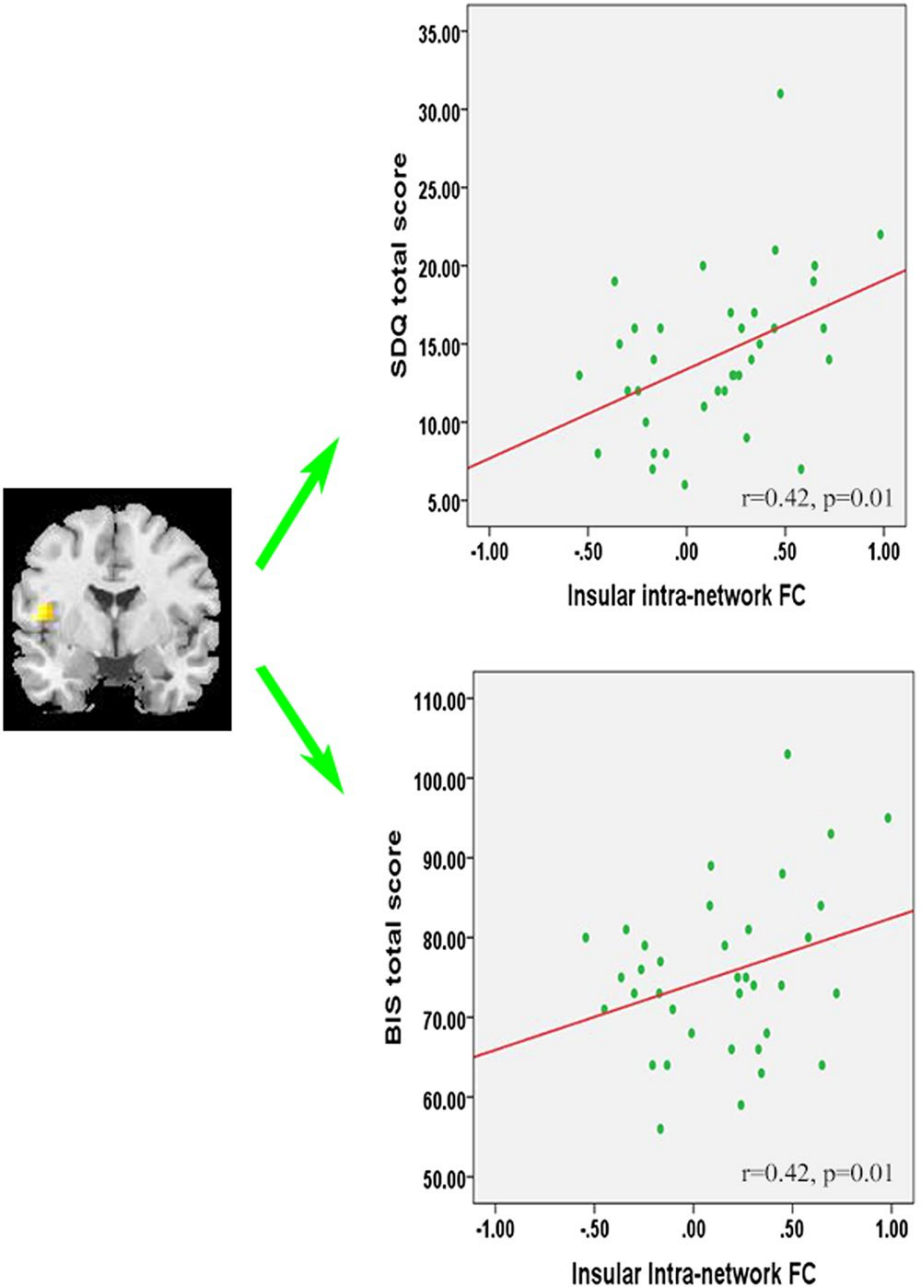
Correlations of Insular Intra-network Functional Connectivity with Clinical Variables in Male Adolescents with Conduct Disorder.

Increased FC in the left AI (belonging to the frontothalamic–basal ganglia network) was correlated positively with SDQ (*r* = 0.42; 95% CI, 0.11–0.69) and BIS total scores (*r* = 0.42; 95% CI, 0.11–0.64) (Fig. 3), as well as BIS nonplanning (*r* = 0.40; 95% CI, 0.06–0.66) and motor (*r* = 0.38; 95% CI, 0.06–0.60) subscale scores, in patients with CD (Supplemental Figure S2). Moreover, higher level of CU traits was associated with greater reduction in anti-correlation between FPN and DMN (r = 0.60; 95% CI, 0.32–0.85; Fig. 2C).

Since impulsivity is considered be a continuous variable across normal adolescents and those with behavioral problems^36^, correlation analysis of the BIS data from all participants were performed and showed that increased intra-network FC in the AI (frontothalamic–basal ganglia network), PrCG (right frontoparietal network), and PoCG (temporal/limbic/visual network) was correlated positively with BIS subscale scores (Supplemental Table S2).

We also did a FDR correction on the correlation findings (with a significant threshold at q < 0.05). The results with FDR correction were almost identical to the prior findings. In the patient group, the correlation of insular FC with SDQ total score still remained significant (q = 0.04), while those with BIS total score, BIS non-planning (BIS-NP) and motor (BIS-M) subscale scores all showed trends of significance (q = 0.11 for all). Across two groups, the results showed significant correlations of insular FC with BIS total score, BIS-NP score and BIS-M score (q < 0.00 for all); meanwhile, PrCG FC also showed a significant correlation with BIS-M score (q = 0.02) and a trend of significance with BIS total score (q = 0.09). For inter-network FC, the significant correlation of FPN-DMN FC with CU traits still survived after FDR correction (q < 0.001).

## Discussion

To our knowledge, this study is the first to explore the relationship between intrinsic brain functional architecture and psychopathic traits in individuals with conduct problems. Our findings showed that adolescents with CD exhibited increased FC within distributed brain networks including frontothalamic–basal ganglia network, right fronto-parietal network and temporal/limbic/visual network, which are suggested to be associated with reward, response inhibition and sensory information processing respectively^52^. Furthermore, we also observed decreased anti-correlation between the FPN and DMN, which have been established to subserve cognitive control and internal thought, respectively^56^. Most interestingly, the present study revealed that the heightened couplings within brain networks were consistently associated with the impulsivity traits, while the weakened interaction between brain networks may underpin the CU traits in adolescent CD patients.

### Weakened interaction between Intrinsic Networks Associated with CU Traits

Consistent with our hypothesis, the present study revealed that reduced FPN-DMN anti-correlation related to high level of CU traits (i.e., lack of empathy) in CD patients. Empathy, an ability to understand and share the mental states of others, has been reported to be associated with the DMN^57–59^. Several core regions in the FPN, such as the anterior cingulated cortex, anterior insula and right temporoparietal conjunction, have also been reported to be involved in empathy^57, 60, 61^. Of note, a recent resting-state fMRI study demonstrated a significant association of the FPN-DMN anti-correlation with trait emotional intelligence^15^. Furthermore, by applying a social working memory paradigm, Xin *et al*.^14^ found that greater reduction of the FPN-DMN anti-correlation related to higher empathic ability in healthy subjects. The evidence together suggests that the DMN and PFN activity, as well as their interaction, are all important for empathy. The current findings shed further light on the impaired FPN-DMN interaction and its contribution to the CU traits (i.e., lack of empathy) in patients with conduct problems^14, 15^. Our finding also concurs with a recent resting-state fMRI study showing disrupted functional integration between the DMN and attention control networks in a population with antisocial personality disorder^62^, suggesting that the imbalance between FPN and DMN activity may be sustained from the adolescents with CD to its severe form adult psychopathy. Future longitudinal study is strongly warranted to verify this hypothesis.

### Heightened Coupling within Brain Networks Associated with Impulsivity Traits

Impulsive behaviors have been proposed to be arisen from two separate processes –increased sensation seeking in terms of pursuing rewards and deficit of motor or response inhibition^20^. The present study reveal that CD is involved in both neuropathological aspects of impulsivity, as evidenced by our findings of the associations of impulsivity with heightened activity in the right fronto-parietal and temporal/limbic/visual network both relevant to response inhibition, as well as the frontothalamic–basal ganglia network subserving reward processing^52^ in this disorder.

We found that hyper-connectivity within the frontothalamic–basal ganglia network located specifically in the VmPFC and AI, both of which are known to be critical in the computation of reward expectations^3^. Previously, increased VmPFC and AI activation in response to punished reversal errors studies have been documented in children with CD^6^ and offenders with psychopathy^63^. Hyper-function in these regions has been linked to impaired ability to perceive violation of reward expectations (when reward is expected but punish is received), which may lead to frustration and, in turn, give rise to impulsive decision-making and reactive aggression^3^. Consistent with this notion, our correlation analysis showed significant associations of insular hyper-function with impulsivity traits and conduct problems (as measured by SDQ total score) in CD patients.

Notably, these findings fit nicely with a series of findings that reward-induced decision-making structures, particularly anterior insula and VmPFC, show disruption in CD, but that this disruption is not driven by CU traits^7–9^. Furthermore, a most recent work suggests, consistent with the current findings, that impulsivity symptoms in youth with CD might be driving this dysfunction^64^. This work is also consistent with Rubia’s work in “clean” CD and ADHD samples^4, 5, 27^, suggesting that the “hot” paralimbic system regulating the movitation/reward may characterize the pathophysiology of “pure” CD. Given the consistency of these findings across studies from groups in the US, Europe and China, the functional abnormality in this reward-related frontothalamic–basal ganglia circuitry might be a potentially key treatment target for impulsivity and conduct problems for this mental disease in childhood and adolescence.

Interestingly, we also found enhanced connectivity within the right fronto-parietal network and temporal/limbic/visual network. The hyper-connectivity in the first network was specifically located at the pre-central gyrus adjacent to the frontal eye field but belonged to the supplementary motor area (SMA), and that in the second network was located at the post-central gyrus (a primary somatosensory area) and fusiform. The (pre)motor and sensorimotor cortices have been consistently observed to be involved in motor inhibition in healthy subjects^20^. Particularly, the increased SMA activity has been found to be correlated with poorer stop-signal task performance in healthy subjects^65–67^. In CD patients, our prior work and another study both identified dysfunction in the pre-motor and sensorimotor cortex during response inhibition task^5, 21^. Of note, a recent resting-state fMRI study in healthy subjects found a positive association between impulsivity and FC in the sensory/visual module^17^. However, our study did not observe a significant relationship between the increased fusiform FC and impulsivity. Thus, the role of the disrupted resting-state function of visual cortex in CD psychopathology still need to be examined in future studies.

What should be noted is that amygdale dysfunction was not observed in our data, which is apparently inconsistent with a previous resting-state fMRI study^68^. However, instead of functional connectivity with other brain regions, previous report focused on a different aspect (i.e., energy) of amygdale using the amplitude of low-frequency fluctuation (ALFF). Our findings are also different from two previous reports (n = 18) identifying uncoupling within brain networks, such as the default mode, sensory-motor, or the visual networks^69, 70^. Such differences may be due to the clinical heterogeneity among CD patients^27^. Compared to our relatively larger sample from 36 CD patients with substantial clinical profiles (6 patients were excluded for their excessive head motions, please see the supplementary Text), these previous studies using the same dataset from 18 CD adolescents reported no records on the severity of clinical symptoms and psychopathy. Thus, clinical profiles cannot be directly compared between those studies and ours. However, given the robust evidence that clinical features, such as the severity of CU traits, modify the neuropathological characteristics in CD patients^10^, it is possible that homogeneity of clinical features might mix the findings of resting-state brain network in this population. Moreover, unlike the previous studies, we further excluded comorbidity of ADHD, depression and anxiety, as these three had been proven to have high comorbidity rate with CD and modulate the amygdale activity^27, 71^.

### Limitations

Several caveats of our findings should be mentioned. First, this study assessed the psychopathy across patients and healthy controls using the APSD, which so far has not been established a cutoff score for classification of a high level of psychopathy. Previous studies of adolescents have used median splits (e.g., > 11 for males, 9 for females)^72^, cutoff scores (such as a score of 20)^6, 11, 73^, or percentile rankings (e.g., the top 33%)^74^ to classify the level of psychopathy. If using a stringent cutoff like a score of 20, this study had only three patients with APSD scores > 20. Thus, future studies are warranted for samples with more severe psychopathy. However, it has also been suggested that using an extreme groups approach may result in relatively limited number of samples and limited variability of CU traits, raising the possibility of Type II error in the correlation analysis between neural deficits and psychopathic traits^75^. Given the significant differences of APSD total and CU traits scores between CD patients and HC (p < 0.003 and 0.005, respectively), it makes sense to investigate the neural correlates of psychopathy in this CD sample with large variations of CU traits scores. Second, our comparison group showed relatively higher APSD total scores comparing to previous studies from the US^11, 76, 77^, which may reduce the statistical significance in comparing resting-state networks between two groups. Since we have carefully excluded subjects with any psychiatric or neurological disease in HC group by two experienced psychiatrists, future studies, particularly for Chinese sample, should keep in mind on this issue to verify whether the average level of APSD scores of Chinese adolescents is higher relative to western world. Third, we did not record the illness duration of the CD patients, which might be a potential confounding factor. Forth, by using a FDR correction on our correlation findings, it is interesting to note that the correlation of insular FC with BIS scores survived in two groups, but showed a trend of significance in the patient group. A possible reason is that our sample with 36 patients is not large enough to detect the statistic significance of this correlation at a stringent threshold. Future studies with larger samples are needed to verify the associations of resting-state insular dysfunction with impulsivity traits. Finally, as resting-state network did not explicitly engage any cognitive tasks, further examination of FPN-DMN dynamic interaction in CD patients during tasks such as decision making in social context may reveal dysfunction in more specific dimension of the interplay between the executive function and the social understanding to others.

In summary, while current theories of biological substrates of psychopathic traits in CD are predominantly arisen from task-driven fMRI approaches focusing on functional alterations in specific brain regions, the present data raise the possibility that more global network dysfunctions may also be underpin the core psychopathic traits in this disorder. Critically, our study provides initial evidence that the FPN-DMN anti-correlation is associated with the CU traits. Our observations underscore the importance of spontaneous dynamic among large-scale networks in the mechanism of psychopathic traits in CD, and raise the question whether restoring this diminished competition between core intrinsic networks should be a novel treatment target for focused training, neuromodulation or pharmacotherapy approaches that purport to overcome CU traits – one of the most persistent challenges in treating conduct problems.

## Electronic supplementary material

Supplementary Information
