# Supplementary material for: Alterations of Brain Functional Architecture Associated with Psychopathic Traits in Male Adolescents with Conduct Disorder
Source: Sci Rep. 2017 Sep 12;7:11349. doi: 10.1038/s41598-017-11775-z (PMC5595864; doi:10.1038/s41598-017-11775-z)
Supplement: Supplementary file 1 — Supplementary Information [file 41598_2017_11775_MOESM1_ESM.doc]

**Supplemental Materials for**

**Alterations of Brain Functional Architecture Associated with Psychopathic Traits in Male Adolescents with Conduct Disorder**

**Weidan Pu ^1,2^, MD, PhD, Qiang Luo^3,4^, PhD, Yali Jiang ^1,2^, PhD, Yidian Gao ^1,2^, PhD, Qingsen Ming ^1,2^, PhD, Shuqiao Yao ^1,2^,*, MD, PhD**

*** Correspondence author: e-mail:** [**shuqiaoyao@163.com**](mailto:shuqiaoyao@163.com)**.**

**Text S1.Functional magnetic resonance imaging data acquisition and preprocessing**

Resting-state functional magnetic resonance imaging (fMRI) data were acquired using the following parameters: repetition time = 2000 ms, echo time = 30 ms, flip angle = 90°, matrix = 64×64, slice thickness = 4 mm, gap = 0 mm, and number of slices = 36. A standard head coil was used for radiofrequency transmission and reception of the nuclear magnetic resonance signal. Foam pads and ear plugs were used to minimize head motion and scanner noise. All subjects were instructed to keep their eyes closed, to think about nothing in particular, and to move as little as possible. Two hundred and six timepoints were acquired; 10 initial images were discarded to allow for scanner stabilization and subjects’ adaptation to the environment. Data preprocessing was then conducted using SPM5 (http://www.fil.ion.ucl.ac.uk/spm) and DPARSF (Data Processing Assistant for resting-state fMRI software, http://www.restfmri.net). The remaining functional scans were quickly corrected for within-scan acquisition time differences between slices, and then realigned to the middle volume to correct for interscan head motion. Each participant’s interscan motion was assessed with translation/rotation, and scans with translation > 1 mm or rotation > 1^o^ in any direction were excluded. Data from six patients with conduct disorder (CD) and three healthy controls (HCs) who met these criteria were excluded from further analysis. Thus, scans from the remaining 36 patients with CD patients and 38 HCs were spatially normalized to a standard template (Montreal Neurological Institute), resampled to 3 mm^3^, and spatially smoothed using a 6 × 6 × 6-mm full width at half maximum Gaussian kernel. There were no significant differences between the excluded patients and HCs as to age, sex and IQ.

**Figures/Tables**

**Figure S1. Fifteen independent Components across Patients and Healthy Controls ^a^**

**
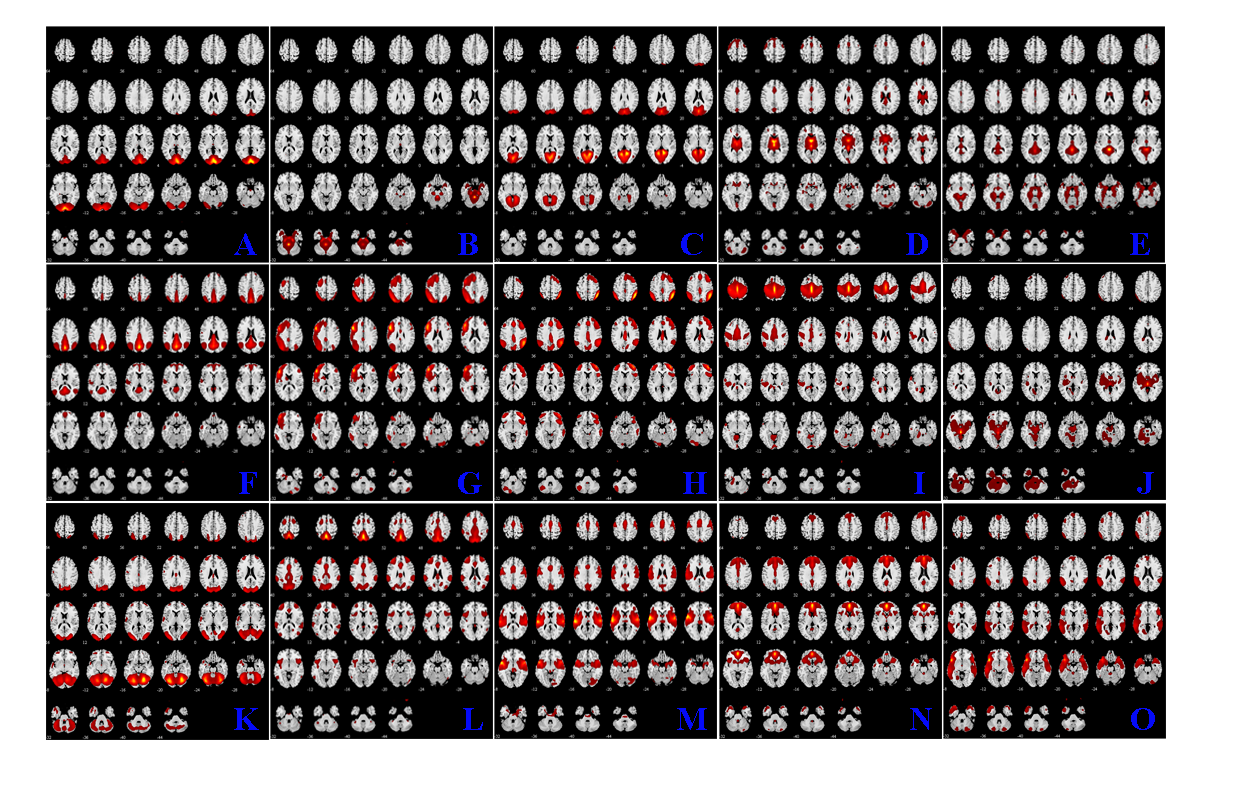
**

^a^ These independent components corresponds to the Table S1: (A) Cebebellum/visual network, (B) Cebebellum/midbrain network, (C) Occipital network, (D) Fronto-thalamus-basal ganlia network, (E) Fronto-temporal/para-limbic network, (F) Posterior default mode network, (G) Left fronto-parietal network, (H) Right fronto-parietal network, (I) Sensorimotor network, (J) Cerebellum/midbrain network, (K) Temporal/limbic/visual network, (L) left fronto-parietal network, (M) Transverse temporal network, (N) Default-mode network, (O) Fronto-temporal/limbic network.

**Figure S2. Correlations of Insular Intra-network FC with Sub-scores of Barratt Impulsiveness Scale in Male Adolescents with Conduct Disorders ^a^**

**
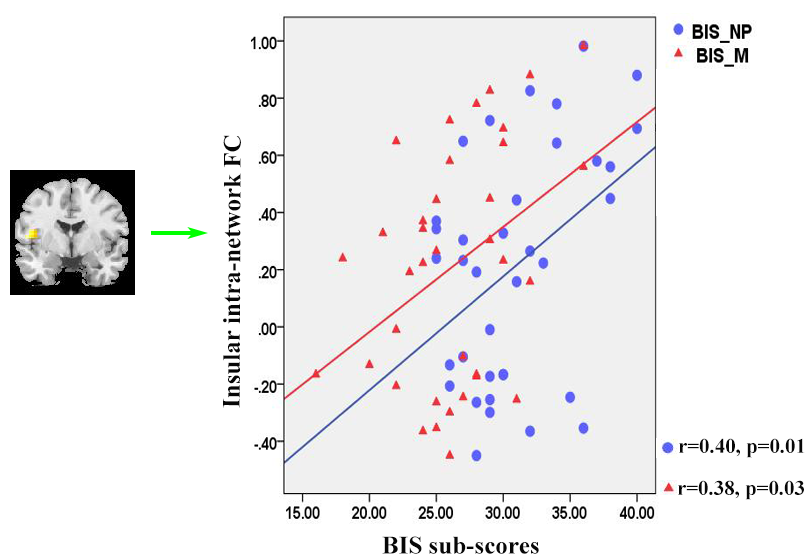
**

^a^ BIS: Barratt Impulsiveness Scale, BIS_NP: unplanned impulsiveness sub-score of Barratt Impulsiveness Scale, BIS_M: motor impulsiveness sub-score of Barratt Impulsiveness Scale.

**TABLE S1. Voxel-based Spatial Correlation with Larid et al 2011 (1) Brain Maps**

| Component Number | Network | ICN(1) | Pearson’s r | Function(1) |
| --- | --- | --- | --- | --- |
| IC6 | Cebebellum/visual | 11 | 0.47 | Higher level visual processing |
| IC8 | Cebebellum/midbrain | 5 | 0.18 | Sensorimotor, interoception |
| IC9 | Occipital | 12 | 0.66 | Visual processing |
| IC11 | Fronto-thalamus-basal ganlia | 3 | 0.45 | Reward processing, interoception and inhibition |
| IC12 | Fronto-temporal/para-limbic | 1 | 0.26 | Emotional processing |
| IC14 | Posterior default-mode | 13 | 0.35 | Theory of mind/social recognition |
| IC15 | Central executive | 18 | 0.38 | Senior cognitive processing, executive function, working memory, control |
| IC17 | Right fronto-parietal | 15 | 0.27 | Attention, inhibition and memory |
| IC19 | Sensorimotor | 9 | 0.43 | Motor execution and learning |
| IC21 | Cerebellum/midbrain | 5 | 0.14 | Sensorimotor, interoception |
| IC22 | Temporal/limbic/visual | 14 | 0.23 | Sensorimotor, autonomic and cognitive functions |
|  |  | 11 | 0.17 | Higher level visual processing |
| IC26 | Left fronto-parietal | 4 | 0.17 | Executive function, cognitive control and inhibition |
|  |  | 7 | 0.13 | Reasoning and visuospatial processing |
| IC27 | Transverse temporal | 16 | 0.40 | Language and auditory processing |
| IC28 | Anterior default-mode | 2 | 0.25 | Theory of mind, self-reflection, autobiographical memory |
| IC29 | Fronto-temporal/limbic | 1 | 0.17 | Emotion/interception processing |

**TABLE S2. Significant Correlations of Impulsivity with Altered Functional Connectivity across Patients and Healthy Controls** a

|  | BIS_total | | | BIS_NP | | | BIS_M | | | |
| --- | --- | --- | --- | --- | --- | --- | --- | --- | --- | --- |
|  | r | p | 95% CI^a^ | r | p | 95% CI | r | p | | 95% CI |
| INS | 0.49 | 0.000 | 0.27, 0.64 | 0.40 | 0.001 | 0.17, 0.57 | - | | - | - |
| PrCG | 0.24 | 0.04 | 0.28,0.43 | - | - | - | 0.31 | | 0.009 | 0.14, 0.48 |
| PoCG | - | - | - | - | - | - | 0.33 | | 0.004 | 0.12, 0.51 |

^a^ 95% CI:biased corrected and accelerated 95% confidence intervals.

BIS_total: BIS: Barratt Impulsiveness Scale total score; BIS_NP: unplanned impulsiveness sub-score of Barratt Impulsiveness Scale; BIS-M: motor impulsiveness sub-score of Barratt Impulsiveness Scale; AI: anterior insula; PreCG: precental gyrus; PoCG: postcentral gyrus.
